# Acute Consumption of Bordo Grape Juice and Wine Improves Serum Antioxidant Status in Healthy Individuals and Inhibits Reactive Oxygen Species Production in Human Neuron-Like Cells

**DOI:** 10.1155/2018/4384012

**Published:** 2018-03-01

**Authors:** Cristiane Copetti, Fernanda Wouters Franco, Eduarda da Rosa Machado, Marcela Bromberger Soquetta, Andréia Quatrin, Vitor de Miranda Ramos, José Cláudio Fonseca Moreira, Tatiana Emanuelli, Cláudia Kaehler Sautter, Neidi Garcia Penna

**Affiliations:** ^1^Department of Food Technology and Science, Center of Rural Sciences, Federal University of Santa Maria (UFSM), 1000 Roraima Avenue, 97105-900 Santa Maria, RS, Brazil; ^2^Department of Chemical Engineering, Center of Technology, Federal University of Santa Maria (UFSM), 1000 Roraima Avenue, 97105-900 Santa Maria, RS, Brazil; ^3^Department of Biochemistry, Center of Oxidative Stress Research (CEEO), Federal University of Rio Grande do Sul (UFRGS), 2600 Ramiro Barcelos Street–Annex, 90035-003 Porto Alegre, RS, Brazil

## Abstract

Few studies investigated the biological effects of American grape cultivars. We investigated the metabolic response after acute consumption of grape juice or wine from Bordo grapes (*Vitis labrusca*) in a placebo-controlled crossover study with fifteen healthy volunteers. Blood samples were collected 1 hour after the intake of 100 mL of water, juice, or wine to measure TBARS, ABTS, FRAP, glucose, and uric acid levels. To evaluate differences in cellular response, intracellular reactive species production (DCFH-DA) and metabolic mitochondrial viability (MTT) were assessed after exposure of human neuron-like cells (SH-SY5Y) to juice or wine. Glycemia was reduced after juice or wine consumption, whereas blood levels of uric acid were reduced after juice consumption but increased after wine consumption. Juice and wine consumption reduced plasma lipid peroxidation and increased plasma antioxidant capacity (ABTS and FRAP assays). Furthermore, juice inhibited H_2_O_2_-induced intracellular production of reactive species (RS) and increased the viability of SH-SY5Y cells. In contrast, wine (dealcoholized) exhibited a per se effect by inducing the production of RS and reducing cell viability. These results indicate a positive impact of acute consumption of Bordo juice and wine on human oxidative status, whereas only juice had protective effects against oxidative stress-induced cytotoxicity.

## 1. Introduction

Oxidative stress is caused by the insufficient capacity of biological systems to neutralize the excessive production of reactive species [1], which leads to oxidative damage in cells. Neuronal cells are particularly susceptible to reactive oxygen species (ROS) and reactive nitrogen species (RNS) due to their high metabolic activity, low antioxidant capacity, and their nonreplicative nature. Furthermore, the abundance of mitochondria in brain cells increases the generation of reactive species [2].

Fruits and vegetables have many bioactive compounds such as polyphenols, which have antioxidant properties with a role in the protection of cellular macromolecules against oxidative damage induced by ROS and RNS [3–5]. There is increasing evidence that polyphenols may protect cell constituents against oxidative damage and, therefore, limit the risk of various degenerative diseases associated with oxidative stress [6]. Studies have repeatedly shown an inverse association between the risk of several chronic human diseases and the consumption of polyphenol-rich diet [7]. The phenolic group of polyphenols can accept an electron to form relatively stable phenoxyl radicals, thereby disrupting chain oxidative reactions in cellular components. It is well established that polyphenol-rich foods and beverages may increase plasma antioxidant capacity [8, 9].

Grapes contain high levels of polyphenols, which have been demonstrated to reduce oxidative stress, inflammatory response, and the oxidation of low density lipoprotein cholesterol (LDL-c), while inhibiting platelet aggregation and improving protection against atherothrombotic episodes. Such actions promote beneficial effects on coronary heart disease (CHD) and atherosclerosis [10–12]. Red wines are rich in polyphenols, such as phenolic acids (gallic acid, caffeic acid, *p*-coumaric acid, and others), stilbenes (*trans*-resveratrol), and flavonoids (catechin, epicatechin, quercetin, rutin, myricetin, and others) [13]. Therefore, a regular consumption of red wine has been linked with the “French paradox,” which explains the apparent compatibility of a high-fat diet with a low mortality from CHD. Also, current evidence suggests that wine consumption is correlated with a reduction in the incidence of neurodegenerative diseases associated to oxidative stress such as Alzheimer's and Parkinson's disease [14]. Grape juice is a natural and nonalcoholic beverage that contains sugars, minerals, and phenolic compounds like anthocyanins, among which malvidin 3,5-diglucoside is the major one [15]. This beverage has been shown to exert antioxidant activity in vitro and in vivo, as well as hypolipidemic and anti-inflammatory effects in rats and humans [16–18].

However, few studies have compared the effects of wine and juice consumption in biological parameters of humans, and these studies used European grape species (*Vitis vinifera*) [19–21]. In contrast, the biological effects of wine and juice from American grape species (*Vitis labrusca*) have not been compared. This investigation is particularly interesting as the red grape cultivar “Bordo” (*V. labrusca*), which is the most important grape cultivated in Brazil [15], has been recently demonstrated to exhibit higher content of phenolic compounds and *in vitro* antioxidant capacity than *V. vinifera* species [22]. In the present study, we compared the biological effects of juice and wine from “Bordo” grapes (*V. labrusca* L) by assessing blood antioxidant response after human consumption and the oxidative cellular response in human neuron-like cells (SH-SY5Y).

## 2. Materials and Methods

### 2.1. Bordo Grape Juice and Wine

The commercial samples of Bordo grape juice and Bordo wine were produced by a winemaker (Casa Perini, Farroupilha, RS, Brazil). The grape fruits used to prepare juice and wine were harvested in Farroupilha (29° 13′ 30″ S, 51° 20′ 52″ W, altitude 783 m), in the State of Rio Grande do Sul, Brazil, on January 2014. Bordo grape juice was prepared by the enzymatic method, in which grape is crushed and then heated to at least 65°C in a hot macerator. Next, commercial pectolytic enzymes are added and must be kept between 55°C and 60°C during 1-2 h. The extracted juice is then clarified, pasteurized, and bottled [23]. Bordo wine was obtained from vinification process by the coupled dispositive to the crushing machine that is called dewaxing. In winemaking of red wine, grape skin remains inside tanks during fermentation for extraction of anthocyanin pigments [24].

### 2.2. Determination of Bioactive Compounds in Bordo Juice and Wine

The total phenolic content was determined at 760 nm using the Folin–Ciocalteu method and gallic acid as standard [25]. Total anthocyanin content was assessed at 520 nm as the difference of absorbance before and after sample decoloration using sodium bisulfite at pH 0.8 and was expressed as mg of malvidin-3-glucoside/L [26]. The total flavonoid content was estimated at 510 nm using a standard curve of catechin (0–200 mg/L) [27].

### 2.3. Antioxidant Capacity of Bordo Juice and Wine

The antioxidant capacity of grape juice and red wine were determined using the 2,2′-azino-bis (3-ethylbenzothiazoline-6-sulphonic acid) (ABTS) and ferric reducing antioxidant power (FRAP) methods as described by Re et al. [28] and Benzie and Strain [29], respectively. The ABTS assay is assessed at 764 nm and is based on the ability of the sample to scavenge the cation radical ABTS^·+^. The FRAP assay is assessed at 620 nm and is based on the reduction of ferric-tripyridyltriazine (Fe III-TPTZ) by antioxidants present in the samples forming ferrous-tripyridyltriazine (Fe II-TPTZ), a blue-colored product. Trolox was used in the calibration curve.

### 2.4. In Vivo Study

#### 2.4.1. Participants

The study design was approved by the Ethics Committee of Federal University of Santa Maria (CAAE 39197614.3.0000.5346), and all subjects signed a written agreement before participating. Fifteen healthy volunteers, with mean age 24.0 ± 3.6, were recruited from the University staff. The health status and medical history of volunteers were examined by a structured interview for inclusion or exclusion according to the criteria shown in Table 1.

#### 2.4.2. Study Design

**Figure 1:**
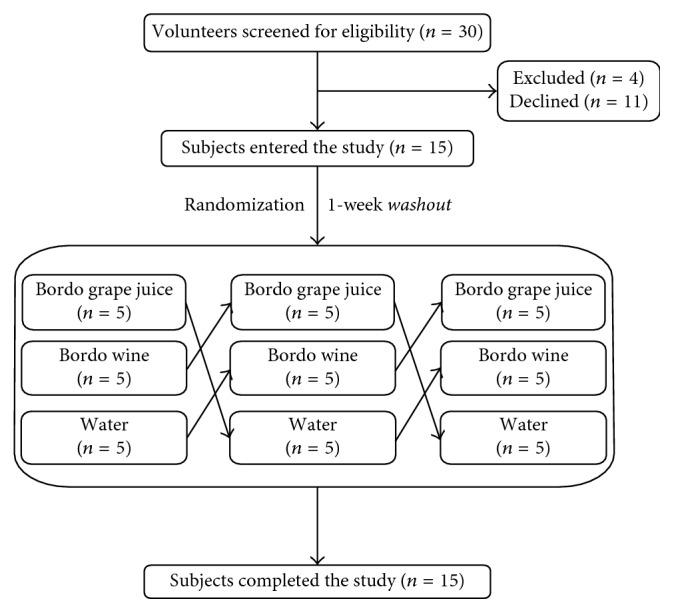
Flowchart of the selection of subjects in the controlled intervention study.

In this crossover-controlled clinical study, 15 volunteers were included, 10 women (67%) and 5 men (33%). All participants received the three treatments, namely, Bordo grape juice, Bordo wine, and water (control) with a washout period of 1 week between treatments. The sequence of the treatments was randomized among the participants as depicted in Figure 1.

Participants were oriented to follow a low-antioxidant diet for 48 h prior to the day of intervention, avoiding some fruits, vegetables, and juices, mainly rich in anthocyanins, tea, coffee, cocoa foodstuffs, and alcoholic beverages. This dietary restriction was aimed to reduce dietary phenolic compounds from blood as these compounds are typically cleared within 48 h of consumption [30]. The intake of energy, macronutrients, dietary fiber, and antioxidants before the intervention was monitored using a prospective 48 h dietary record. Each participant served as his own control because we compared data obtained after either juice, wine, or water consumption with the respective baseline values before consumption. In the day of intervention, baseline blood samples were collected after overnight fasting (12 h), then subjects consumed 100 mL of Bordo grape juice, Bordo wine, or water. One hour after drinking, test blood samples were collected. This protocol was chosen based on a previous study that revealed maximal antioxidant capacity and phenolic concentration in serum 1 h after the intake of the fruit or beverage [30]. No food was provided during this period.

#### 2.4.3. Blood Collection and Analyses

Fasting venous blood samples were collected through aseptic venipuncture into heparinized tubes and EDTA-containing tubes that were centrifuged (1500 ×g, 10 min) to yield plasma for thiobarbituric acid reactive species (TBARS), ABTS, and FRAP analysis. Blood collected in tubes without additives was centrifuged (1500 ×g, 10 min) to yield serum for analysis of uric acid and glucose. Serum and plasma samples were stored at −80°C until analysis.

Uric acid and glucose were determined in serum using commercially available enzymatic kits (Bioclin, Belo Horizonte, Brazil). Lipid peroxidation was determined by measurement of TBARS at 535 nm in plasma [31]. The antioxidant capacity of plasma was assessed by the ABTS [28] and FRAP assays [29].

### 2.5. Cell Culture Assays

Human neuron-like cell line SH-SY5Y obtained from the European Collection of Authenticated Cell Cultures (ECACC) were maintained in 75 cm^2^ flasks containing DMEM/F12 medium (1 : 1) supplemented with 10% fetal bovine serum (FBS) and 1× antibiotic/antimycotic solution (Sigma-Aldrich). Cells were cultured in a humidified incubator set at 37°C with 5% CO_2_. When cultures reached confluence, cells were trypsinized and seeded at a density of 30 × 10^3^ cells/cm^2^ in 96-well culture plates. Treatments started 24 hours after seeding. All treatments were performed using 1% FBS supplemented medium. Bordo juice and wine were freeze-dried to remove water and alcohol and then dissolved in culture medium at the desired concentration (w/v). Cells were exposed to these juice and wine solutions or vehicle (culture medium).

#### 2.5.1. Determination of Intracellular ROS Production

Intracellular ROS production was detected using the 2′,7′-dichlorofluorescein diacetate (DCFH-DA, Sigma) as described [32]. Cells were pretreated with Bordo juice or wine (solutions in culture medium, Section 2.3) or vehicle (culture medium) during 2 h and then incubated in the absence (control) or presence of H_2_O_2_ (100 *µ*M) for 3 h before monitoring DCF fluorescence. H_2_O_2_ was used as a positive control to induce ROS generation [33]. DCFH-DA stock solution was dissolved in DMSO at a final concentration of 10 mM and stored at −20°C protected from light. Before cells were treated, DCFH-DA was diluted to 100 *μ*M using 1% FBS-supplemented medium solution. After addition of DCFH-DA, cells were incubated at 37°C, with 5% CO_2_, and protected from light exposure for 1 h. After DCFH internalization, the medium was replaced by fresh 1% FBS-supplemented medium solution. When internalized, ROS cause DCFH oxidation, and it becomes a fluorophore (DCF), which was quantified using a SpectraMAX i3 (Molecular Devices) fluorescence plate reader (Ex/Em  =  485/532 nm). Fluorescence was monitored, and the area under the curve (AUC) of fluorescence versus time was calculated.

#### 2.5.2. Metabolic Mitochondrial Viability

Metabolic mitochondrial viability was assessed by the MTT (3-(4,5-dimethylthiazol-2-yl)-2,5-diphenyl tetrazolium bromide) assay as previously described [34]. SH-SY5Y cells were plated onto 96-well plates and exposed to Bordo juice or wine (solutions in culture medium, see Section 2.3) or vehicle (culture medium) during 24 h. Parallel sets of wells were run in the absence or presence of H_2_O_2_ (100 *µ*M) (co-exposure scheme with juice/wine), which was used as a positive control to induce cell death [35]. Then, cells were incubated with MTT for 45 min at 37°C in a humidified 5% CO_2_ atmosphere. The medium was then removed, and plates were shaken with DMSO for 30 min. The optical density of each well was measured at 550 nm (test) and 690 nm.

### 2.6. Statistical Analysis

All the analyses were performed in triplicate. Results were analyzed using the Statistica software package (StatSoft Inc., Tulsa, Okla, USA) and expressed as mean ± SEM. The parameters of the juice and wine were compared by the Student's *t*-test. The effects of wine, juice, and water intake on blood parameters were evaluated by the paired *t*-test to compare baseline versus test data (intragroup comparison) and by analyses of variance followed by Tukey's test for intergroup comparison. Significance was set at *p* < 0.05.

## 3. Results

### 3.1. Characteristics of the Subjects

General characteristics of the study group are presented in Table 2. Fifteen apparently healthy individuals, 5 men and 10 women, respectively, with mean age 24.1 ± 3.7 and body mass index of 23.7 ± 3.2 kg/m^2^ were included. The systolic and diastolic blood pressures of participants were within the intervals of optimal and normal blood pressures according to the Brazilian Society of Hypertension, Brazilian Society of Cardiology, and Brazilian Society of Nephrology [36] and according to US-American Hypertension Guideline [37].

### 3.2. Bordo Grape Juice and Wine Antioxidant Activity In Vitro

The chemical composition of Bordo grape juice and wine in the same serving size (portion) administered to healthy individuals in this study is shown in Table 3. Grape juice and wine showed high amounts of total phenolic content, but wine had higher amount than grape juice (Table 3, *p* < 0.05). The concentration of total monomeric anthocyanins and total flavonols was also higher in wine compared with grape juice (Table 3, *p* < 0.05).

The antioxidant activities were elevated in the two grape beverages used in this study. Bordo wine showed higher antioxidant capacity by the ABTS method, determined by the decolorization of the ABTS^·+^, through measuring the reduction of the radical cation as the percentage inhibition of absorbance at 734 nm, when compared with grape juice (Table 3, *p* < 0.05). On the other hand, the wine antioxidant capacity assessed by the FRAP method, based on the ferric ion reduction (Fe^+3^) capacity, did not differ from juice (Table 3, *p* < 0.05).

### 3.3. Acute Consumption of Bordo Juice and Wine

**Figure 2:**
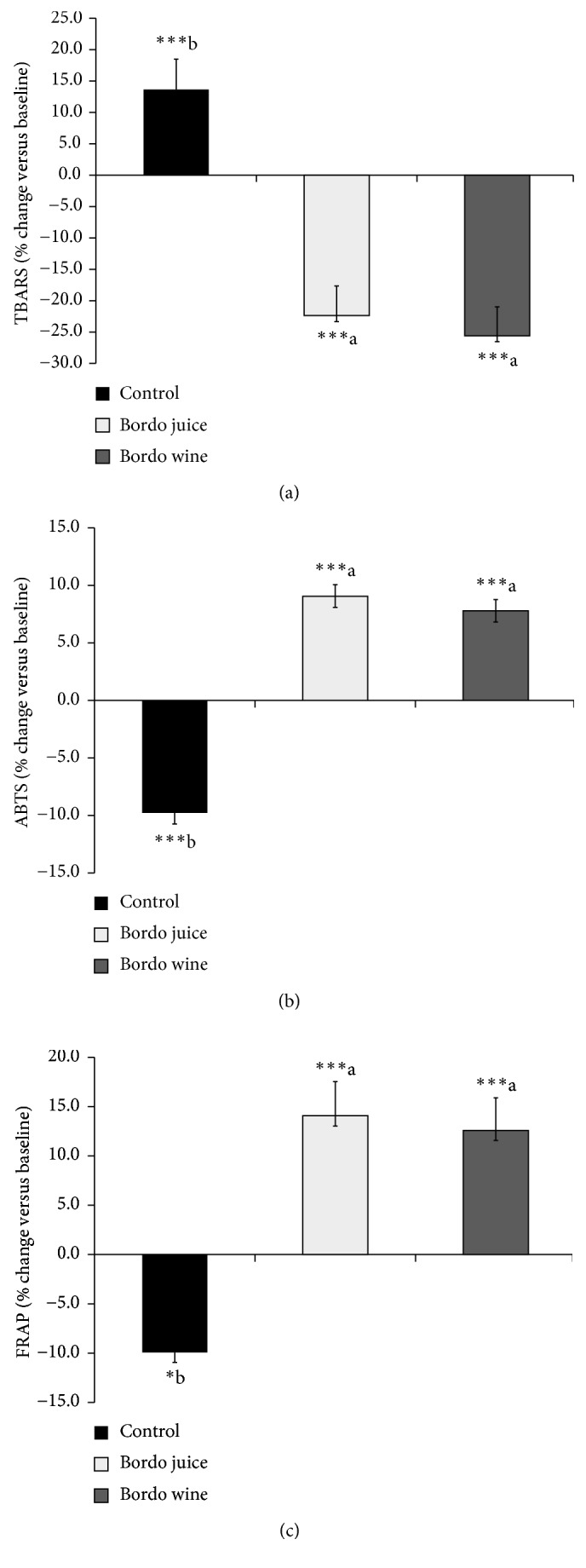
Changes in serum TBARS levels (a) and plasma antioxidant capacity assessed by the ABTS (b) and FRAP (c) assays in humans after consumption of Bordo juice, Bordo wine, or water (control). Results are expressed as percentage of baseline values for each group (means ± SEM, *n* = 15). ^∗^Significantly different from baseline (paired Student's *t*-test; ^∗^*p* < 0.05, ^∗∗^*p* < 0.01, and ^∗∗∗^*p* < 0.001). ^a,b^Different letters indicate significant difference among interventions (Tukey's test, *p* < 0.05).

After the consumption of Bordo grape juice and wine, serum levels of TBARS were, respectively, decreased by 22.3% and 25.7% compared with baseline values (*p* < 0.001), but no significant differences were observed between Bordo juice and wine (Figure 2). Changes in TBARS levels after juice and wine intake were significantly different from changes observed after water intake (*p* < 0.05), which increased (13.6%) TBARS levels compared with baseline values (*p* < 0.001).

A significant increase in the antioxidant capacity levels, measured by ABTS and FRAP assays, was found 1 h after the consumption of Bordo juice (9.1% and 14.1%, resp.) and wine (7.8% and 12.5%, resp.), compared with baseline values (Figures 2 and 2; *p* < 0.05). Changes in ABTS and FRAP levels after juice and wine intake were significantly (*p* < 0.05) different from changes observed after water intake, which decreased ABTS (9.7%; *p* < 0.001) and FRAP values (9.8%; *p* < 0.05) compared with baseline values.

Significant changes were detected in the mean values of serum glucose and uric acid after the intake of the Bordo grape juice and wine (Table 4). Blood glucose was reduced after consumption of Bordo juice and wine compared with baseline values (*p* < 0.01). Furthermore, wine triggered a greater decrease in blood glucose levels compared with water intake (−8.8% versus −2.0%; *p* < 0.05). Consumption of wine had a different effect in blood uric acid levels compared with water and juice (*p* < 0.05; Table 4). Compared with baseline values, blood uric acid levels were increased after the consumption of Bordo grape wine (*p* < 0.05) but decreased after the consumption of water and Bordo grape juice (*p* < 0.01).

### 3.4. Neuroprotective Effects of Bordo Juice and Wine

**Figure 3:**
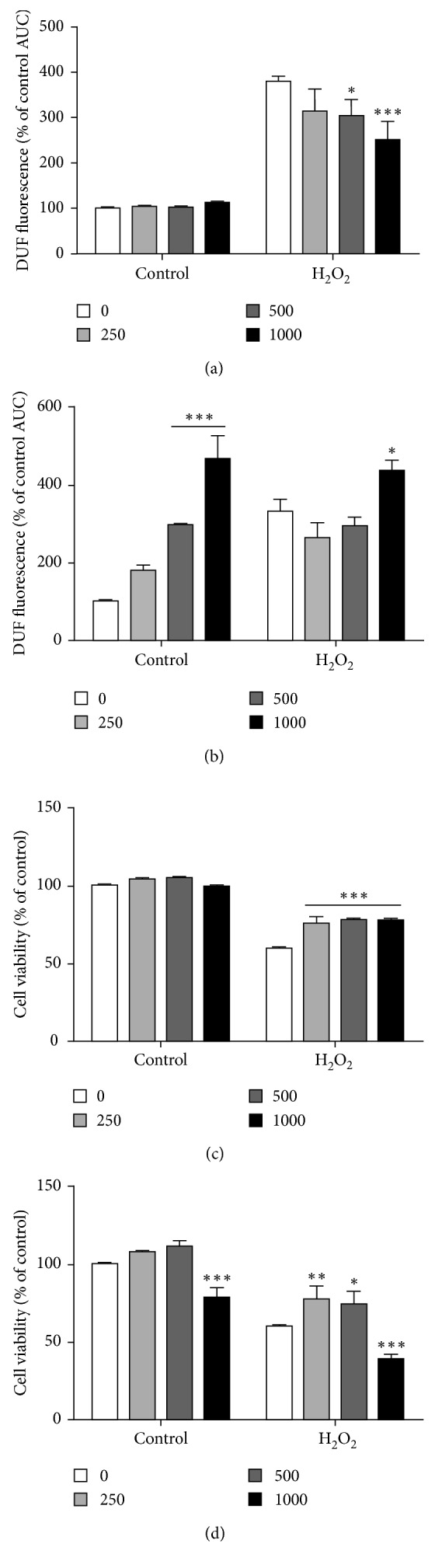
Effect of Bordo grape juice and wine on H_2_O_2_-induced cytotoxicity in SH-SY5Y cells. (a) DCF fluorescence of cells treated with Bordo grape juice. (b) DCF fluorescence of cells treated with Bordo grape wine. (c) Cell viability of cells treated with Bordo grape juice. (d) Cell viability of cells treated with Bordo grape wine. Cells were exposed to 0 (vehicle), 250, 500, and 1000 *µ*g/mL of Bordo juice or Bordo wine during 5 h (panels (a) and (b)) or 24 h (panels (c) and (d)). Two-way ANOVA was applied to all data. ^∗^*p* < 0.05, ^∗∗^*p* < 0.01, and ^∗∗∗^*p* < 0.001 versus the respective vehicle group.

We investigated whether Bordo grape juice and wine could prevent H_2_O_2_-induced intracellular ROS production in SH-SY5Y cells and promote neuroprotective actions (Figure 3). Our results showed that exposure to H_2_O_2_ increased the intracellular ROS production (Figures 3 and 3). However, 500 and 1000 *µ*g/mL of Bordo grape juice significantly (*p* < 0.05 and *p* < 0.001) reduced H_2_O_2_-induced production of ROS (Figure 3), whereas 1000 *µ*g/mL of Bordo grape wine had a prooxidant effect per se by increasing the DCF levels in the absence of H_2_O_2_ (*p* < 0.001; Figure 3). Bordo grape wine was unable to prevent the increase in ROS induced by H_2_O_2_ and only 1000 *µ*g/mL of Bordo grape wine induced further increase in ROS levels compared with H_2_O_2_ (Figure 3, *p* < 0.05).

To determine whether Bordo grape juice and wine could protect against oxidative stress-induced cell death, the SH-SY5Y cell line was used as an in vitro model and H_2_O_2_ as prooxidant insult. After 24 h of H_2_O_2_ exposure in combination with Bordo grape juice, we observed that all tested concentrations (250–1000 *μ*g/mL) of grape juice protected against H_2_O_2_-induced cell death (Figure 3; *p* < 0.001). However, Bordo wine at 1000 *μ*g/mL significantly (*p* < 0.001) reduced cell viability in the absence of H_2_O_2_ (Figure 3). In the presence of H_2_O_2_, only 250–500 *μ*g/mL of Bordo wine protected against cytotoxicity (*p* < 0.01 and *p* < 0.05), whereas 1000 *μ*g/mL of Bordo grape wine induced further cytotoxicity compared with vehicle-H_2_O_2_ (Figure 3, *p* < 0.001).

## 4. Discussion

Polyphenols, which have high antioxidant capacity and exhibit strong protective effect against cellular oxidative damage, are the most abundant secondary metabolites in plants and antioxidants in human diet [38]. Grapes and derivatives contain high amounts of phenolic compounds, mainly flavonoids. In fact, high levels of phenolic compounds were found in samples of Bordo grape juice and wine used in the present study, which may contribute to the high antioxidant potential of those beverages. Furthermore, many of these compounds exhibit multiple biological activities, and these functions are mainly attributed to their antioxidant and antiradical activity [39, 40]. The main finding of our study is that the consumption of Bordo grape juice and wine yielded similar antioxidant effects by increasing total antioxidant capacity and reducing lipid oxidation, despite the higher content of phenolic compounds and in vitro antioxidant activity of Bordo wine compared with juice.

Concerning the study of antioxidant effectiveness, the use of different in vitro models has been recommended, due to the differences between the various free radical scavenging assays [41, 42]. Thus, antioxidant activity of Bordo juice and wine were assessed using the ABTS method, which measures the scavenging of the ABTS radical cation, and the FRAP method, which measures the ability to reduce the ferric-tripyridyl triazine complex (Fe III-TPX) to ferrous complex (FeII-TPZ) under acidic conditions. In our study, the ABTS assay showed significantly higher values compared with FRAP values, mainly for wine. However, the reaction of FRAP method may not be complete even several hours after the initiation of the reaction, mainly because of subsequent dimerizations and polymerizations [43]. Drawbacks of this method are concerned with compounds that have low redox potential and can reduce the Fe III even though they do not behave as antioxidants in vivo [44, 45], interfering compounds that can absorb at the same wavelength and the assay being performed at a nonphysiological pH.

Numerous indices and methods have been used to assess oxidative stress, defined as an imbalance between the production of ROS and their removal by antioxidants. Among various indices, products of lipid peroxidation are the most common group used to evaluate the individual oxidative (antioxidant/prooxidant) status [5, 45]. Lipid peroxidation is a result of complex reactions which yield compounds that can be determined as TBARS [46]. According to García-Alonso et al. [47] a reduction in the lipid oxidation might be associated with the intake of phenolic beverages. Our results showed a significant (*p* < 0.05) decrease in serum lipid peroxidation after the intake of both Bordo juice and wine compared with baseline values, and this effect was not observed after water intake. Similar effects were previously reported in human serum or plasma after the intake of polyphenol-rich foods, and according to these studies, the decrease in lipid peroxidation probably occurred due to the quick absorption of polyphenols into the bloodstream [15, 48, 49]. These phytochemicals are known to prevent lipid peroxidation by scavenging peroxyl radicals [15, 49, 50]. Moreover, evidence from in vitro studies indicates that resveratrol, which is among the most important grape polyphenols [51], can be accumulated into erythrocytes and activates the erythrocyte plasma membrane redox system [52]. Resveratrol may function as an electron donor for this enzymatic system, which reduces extracellular oxidants and recycles oxidized ascorbate, thereby contributing to counteract extracellular oxidative processes [52]. In addition, in silico studies revealed that other grape polyphenols, namely, quercetin, epigallocatechin gallate, catechin, and epicatechin, are able to interact and donate protons to the human NADH-cytochrome b5 reductase, which is a component of the erythrocyte plasma membrane redox system [53]. These mechanisms may underline the antioxidant effect of Bordo juice and wine in serum as observed in the present study.

Short-term studies involving the consumption of polyphenol beverages have reported acute increases in the antioxidant capacity of plasma or serum, which have usually been attributed to the high levels of polyphenolic antioxidants provided by plants [5, 18, 54, 55]. Our findings showed significant (*p* < 0.05) improvement in antioxidant status after the consumption of Bordo grape juice and wine, in opposite to the ingestion of control beverage (water), therefore confirming our hypothesis that polyphenols present in the Bordo juice and wine favorably influence the antioxidant capacity in vivo. Malvidin-3-glucoside (M-3-G), which is the most abundant anthocyanin in grapes and grape products, has similar bioavailability after the ingestion of red wine or dealcoholized red wine, indicating that ethanol in red wine does not seem to affect the absolute uptake and plasma concentrations of M-3-G [56]. Furthermore, increases in plasma anthocyanin concentrations after the consumption of either red wine or dealcoholized red wine were about two times lower than those measured after consumption of red grape juice. These authors did not measure the antioxidant capacity after beverage intake. We found that anthocyanin concentration in Bordo wine was 3 times higher than in Bordo grape juice, but both beverages were similarly effective to increase blood antioxidant capacity and reduce lipid oxidation in humans after consumption.

The hypothesis that flavonoids are responsible for the increase in plasma antioxidant capacity after the intake of flavonoid-rich foods has been disputed by evidence that such an effect could be a consequence of increased uric acid levels [57]. Uric acid has been demonstrated to be one of the major contributors to the antioxidant capacity in human serum [48] and particularly contributes to the antioxidant capacity of serum assessed by the FRAP assay [18, 29]. Fructose from flavonoid-rich fruits has been demonstrated to be responsible for increasing plasma uric acid levels [57]. However, in the present study, the consumption of 100 mL of Bordo juice or wine did not increase glycemia. Moreover, we demonstrated that Bordo grape juice decreased blood uric acid levels, indicating that the increase in antioxidant capacity of serum was promoted by grape juice antioxidants and not by urate. Similar results were recently found after acute consumption of grape juices [18]. On the other hand, we found an increase in serum levels of uric acid after Bordo wine consumption that was parallel to the increase in plasma antioxidant capacity (FRAP and ABTS assays) and to the decrease in plasma lipid oxidation. Similar results were found for port wine consumption [58].

Assays using living cells have proven to be useful for routine testing of various products, producing reliable results for the identification of biological activities, including antioxidant capacity [59]. Excessive ROS production is associated with disruption of cell cycle regulatory mechanisms. In the present study, we used the human neuron-like cells SH-SY5Y, which were challenged with H_2_O_2_ that is among the major physiologically relevant ROS species [60, 61]. Bordo juice inhibited the production of RS and the loss of cell viability induced by H_2_O_2_. In contrast, Bordo wine had only a small protective effect against the loss of cell viability at intermediate concentrations but increased RS production and promoted loss of cell viability per se at the highest concentration. Such an effect was not related to the ethanol content of wine as ethanol was removed by freeze-drying before the experiment.

The direct radical scavenging action of polyphenols requires the presence of the antioxidant at the exact place where such radicals are formed. Polyphenols protect biological membranes from oxidation as they interact with the lipid phase of the membrane with a tendency to incorporate into the outer hydrophilic portion of the phospholipid bilayer [60]. The antioxidant components of fruits and vegetables, such as polyphenols, have been found to possess properties which play a role in protecting cellular macromolecules from ROS-induced damage [3, 4]. Many grape compounds could be responsible for the grape juice antioxidant activity against H_2_O_2_-induced damage in SH-SY5Y cells. Polyphenol composition of wines shows higher complexity when compared with their corresponding juice berries because during the winemaking and maturation processes, there are numerous reactions involving phenolic compounds (enzymatic and chemical oxidation reactions, condensation reactions, hydrolysis, etc.). We propose that wine fermentation process generates compounds that exhibit prooxidant effects at high concentrations and would be responsible for the overproduction of RS induced by the highest wine concentration (1 mg/mL) in SH-SY5Y cells. Conversely, commercial red wine from China exhibited neuroprotective effects against H_2_O_2_-induced oxidative stress in SH-SY5Y cells up to 4 mg/mL [62]. This discrepancy may be attributed to differences in the cultivars used to prepare the wines. Another explanation for the different effect of Bordo wine and juice in the culture assays could be the higher concentration of phenolic compounds in Bordo wine compared with Bordo juice, which could exert a prooxidant effect. In fact, Long et al. [63] showed that addition of phenolic compounds, especially epigallocatechin and epigallocatechin gallate, to the cell culture media rapidly generates substantial amounts of H_2_O_2_. This effect was dose-dependent and significant amounts of H_2_O_2_ (200–400 *µ*M) have been shown to be formed after the addition of phenolics at concentrations ≥ 100 *µ*M.

The small number of individuals studied may be considered a limitation of the present study. However, it should be noted that all the analyses were paired comparisons, which has strong statistical power. In conclusion, the high amount of phenolic compounds found in samples of Bordo grape juice and wine used in the present study may contribute to the high in vitro antioxidant potential of those beverages. Furthermore, the in vitro antioxidant capacity can be reproduced as in vivo antioxidant after acute human intake because the consumption of Bordo grape juice and wine improved antioxidant capacity and reduced lipid oxidation in healthy volunteers. In addition, Bordo juice and wine were able to decrease glucose levels and only wine increased uric acid levels. The same way, wine did not have antioxidant effect in cell culture showing to be toxic at high concentration, whereas juice had antioxidant effects against H_2_O_2_-induced cellular oxidative stress. Bordo grape juice and wine can be used for improving health and as a preventive agent for oxidative stress-related diseases, but wine should be consumed in smaller doses due to the prooxidant effect observed in cell culture.

## Figures and Tables

**Table 1:** Selection criteria of study participants.

Inclusion criteria	Exclusion criteria
Apparently healthy individuals	Pregnant and lactating women
Age 18–35 years old	Alcoholic and smokers
BMI between 18.5 and 29.9 kg/m^2^	Vegetarian diet
SBP < 140 mmHg and DBP ≤ 90 mmHg	Regular use of antioxidants or vitamin supplements
	Chronic diseases (cardiovascular diseases, hypertension, diabetes, liver diseases, cancer, or allergy); gastrointestinal disorders or known metabolic diseases; infections or inflammatory processes visible or known in the three months prior to the study

BMI = body mass index, SBP = systolic blood pressure, and DBP = diastolic blood pressure.

**Table 2:** Baseline characteristics of subjects enrolled in the study.

	Participants (*n* = 15)	
	Male (*n* = 5)	Female (*n* = 10)
Age (years)	23.8 ± 4.0	24.3 ± 4.0
	(19–30)	(22–33)
Weight (kg)	79.0 ± 14.7	61.0 ± 5.8
	(65–95)	(54–70)
Height (cm)	180.6 ± 0.1	160.0 ± 0.1
	(169–191)	(154–172)
BMI (kg/m^2^)	24.3 ± 4.5	23.4 ± 2.5
	(20.1–30.3)	(20.2–28.7)
SBP (mmHg)	117.2 ± 13.6	115.8 ± 9.8
	(110–132)	(100–130)
DBP (mmHg)	81.6 ± 8.2	76.9 ± 4.7
	(70–90)	(70–80)
Practice of physical activity at least once a week (%)	2 (40%)	3 (30%)
Physical inactivity (%)	3 (60%)	7 (70%)

Data are expressed as means ± SEM (minimum–maximum), except for the physical activity/inactivity that was expressed as the number of participants (%). BMI = body mass index, SBP = systolic blood pressure, and DBP = diastolic blood pressure.

**Table 3:** Phenolic composition and in vitro antioxidant activity of the Bordo grape juice and wine.

Parameter	Bordo juice	Bordo wine
Total polyphenol index (*µ*mol GAE/100 mL)	184.2 ± 13.1^b^	371.3 ± 9.6^a^
Total anthocyanins (*µ*mol malvidin-3-glucoside/100 mL)	17.5 ± 22.5^b^	66.74 ± 10.2^a^
Total flavonols (*µ*mol catechin/100 mL)	84.5 ± 9.7^b^	93.6 ± 4.6^a^
Total antioxidant activity		
ABTS (*µ*mol TEAC/100 mL)	316.5 ± 14.6^b^	448.7 ± 12.1^a^
FRAP (*µ*mol TEAC/100 mL)	234.6 ± 9.5^a^	234.9 ± 7.1^a^

Values are means ± SEM of determinations in triplicate. ^a,b^Different superscript letters denote significant differences (Tukey's test, *p* < 0.05). GAE = gallic acid equivalent; TEAC = Trolox equivalent antioxidant capacity.

**Table 4:** Glucose and uric acid levels in healthy individuals at baseline and after the interventions with Bordo grape juice, Bordo wine, and water (control).

Intervention samples			
Biochemical parameters	Control	Bordo grapes	
		Bordo juice	Bordo wine
Serum glucose (mg/dL)			
Baseline	84.1 ± 5.8	74.4 ± 5.5	82.2 ± 7.1
1 h after intervention	82.3 ± 5.5	69.3 ± 6.2	74.8 ± 7.4
Change versus baseline (%)	−2.0 ± 1.5^b^	−6.7 ± 1.7^ab^^∗∗^	−8.8 ± 2.0^a^^∗∗^
Uric acid (mg/dL)			
Baseline	4.8 ± 1.9	4.8 ± 1.6	4.4 ± 1.2
1 h after intervention	4.6 ± 1.8	4.6 ± 1.5	4.6 ± 1.2
Change versus baseline (%)	−4.6 ± 1.4^b^^∗∗^	−4.1 ± 1.1^b^^∗∗^	4.2 ± 1.2^a^^∗^

Results are expressed as means ± SEM (*n* = 15). ^∗^Significantly different from baseline (paired Student's *t*-test; ^∗^*p* < 0.05 and ^∗∗^*p* < 0.01). ^a,b^Different letters indicate significant difference among interventions (Tukey's test, *p* < 0.05).
